# Spatio-Temporal Feature Fusion for Anti-UAV Detection: Integrating Inter-Frame Dynamics and Appearance

**DOI:** 10.3390/s26051492

**Published:** 2026-02-27

**Authors:** Yake Zhang, Xiaoxi Fu, Yunfeng Zhou, Xiaojun Guo, Bei Sun, Yinglong Wang, Yongping Zhai

**Affiliations:** 1 College of Advanced Interdisciplinary Studies, National University of Defense Technology, Changsha 410073, China; zhangyake23@nudt.edu.cn (Y.Z.); fu_xiaoxi@nudt.edu.cn (X.F.); zhouyunfeng24@nudt.edu.cn (Y.Z.); 2 College of Intelligence Science and Technology, National University of Defense Technology, Changsha 410073, China; jeanakin@nudt.edu.cn (X.G.); sunbei08@nudt.edu.cn (B.S.)

**Keywords:** UAV detection, spatio-temporal fusing, YOLO, static detection, motion extract, RK3588

## Abstract

In order to improve the detection capability of low-slow-small UAV targets in complex backgrounds, this paper introduces a novel method that combines spatio-temporal information, which includes (1) an improved YOLO detector for small UAV detection, (2) a motion target detection module, and (3) an integrated combination strategy for static and dynamic judgment. We firstly provided an improved YOLOv11 static detection method by combining SPD Conv, BiFPN and a detect header for high-resolution layers, and then designed a dynamic target-detection algorithm which helps the YOLO method capture minor movement features, finally introducing a fusing strategy of static detection and dynamic judgment. The experimental results on small UAV datasets, including various sky, mountain and building backgrounds, have shown that the proposed approach increases Precision, Recall, and mAP50 by 12.1%, 29.5%, and 29.6%, respectively, compared with the baseline YOLO11 detector. The proposed MSM-YOLO achieves Precision, Recall, and mAP50 of 94%, 92%, and 86.3%, enabling the effective detection of small UAV targets in complex scenarios. Moreover, the ablation experiments also proved the effectiveness of each module. The proposed method was further deployed in a redesigned RK3588 embedded system, achieving 100 fps after optimized process, and it has shown effectiveness and practicality in further air-to-air UAV detection applications.

## 1. Introduction

With the rapid development of artificial intelligence and unmanned aerial vehicle (UAV) technologies, small UAVs have shown wide application in low-altitude economy sectors such as logistics transportation, information reconnaissance, and intelligent transportation. However, while the low-slow-small UAVs, such as consumer-grade drones, play an important role in daily life and industrial production, they also bring new security risks. For instance, incidents of unauthorized flights disrupting aviation operations and privacy infringements caused by drone aerial photography have posed significant challenges to the supervision and prevention of low-altitude security [1,2].

The low-slow-small UAVs usually have a maximum takeoff weight of less than 25 kg and a flight speed below 180 km/h [3], and usually have the characteristics of small size, weak features and fast changes, and presently, there is no completely effective detection method. Taking electro-optical detection as an example [4], using a large field of view (FOV) means the object image size is only a few dozen pixels in a resolution of 1920 × 1080, which results in a significant challenge for deep learning detection methods. Conversely, using a small FOV means the observation area is extremely narrow, and each electro-optical pod can only handle a few UAV targets [5], which is basically unsuitable for object detection when there are multiple UAV targets or other similar objects like birds [6]. Therefore, it is of great significance to detect low-slow-small UAVs from a large view.

**Figure 1:**
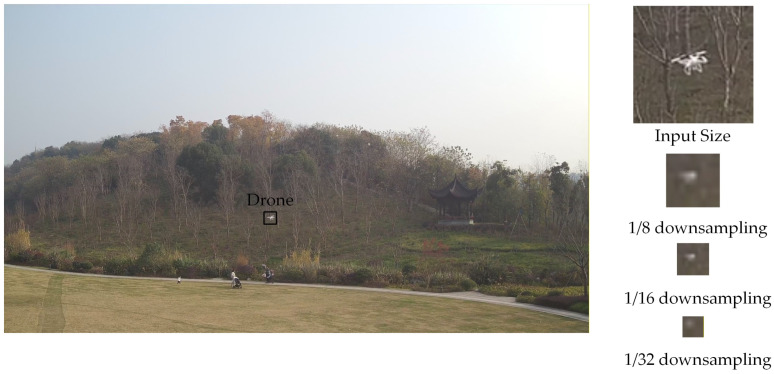
Example of downsampled UAV target size in YOLOv11.

The main difficulties of low-slow-small UAV detection come from the influence of complex environments: on one hand, the objects occupy extremely few pixels in images, which leads to very weak static features of contour and texture. As shown in Figure 1, the pixel proportion of the target is merely 0.005%, far below the generally recognized small targets with a ratio of 0.12%. On the other hand, slow movement or hovering usually results in a small displacement between consecutive frames, which makes it hard to distinguish from motions in the background. Furthermore, the low-altitude sky backgrounds often contain high-complex interference like building edges, vehicles and birds, which result in many false detections. So, considering the extremely small pixel proportion of UAV targets under large view conditions, it is very difficult to detect targets relying solely on single-frame images.

Recently, researchers attempted to improve low-slow-small UAV detection performance based on multi-feature fusion methods. Existing methods [7] include dynamic feature fusing approaches, such as Kalman prediction based on motion trajectory [8], Doppler shift analysis [9], as well as static feature fusing methods like shape-/texture-based CNNs and infrared thermal radiation feature extraction. However, dynamic features [10] generally fail in frequent maneuvers or occlusion scenarios, while the discriminative ability of static features decreases significantly for low-resolution or long-distance targets [11]. Existing methods mostly adopt simple fusion strategies, failing to fully explore the complementarity of spatio-temporal features, and still have significant deficiencies in low-slow-small UAV detection under complex scenarios [12].

Anti-drone detection has attracted increasing attention, and various sensing modalities have been explored beyond purely vision-based approaches. Fang et al. [13] proposed SEB-YOLOv8s for real-time visual detection of unauthorized UAVs, demonstrating the effectiveness of lightweight deep learning models in practical surveillance scenarios.

In addition to visual methods, Brighente et al. [14] introduced ADASS, an embedded audio-based drone surveillance system, while Miesikowska [15] and Lee et al. [16] further investigated UAV classification and identification using acoustic signals under realistic environmental conditions. These studies highlight the potential of audio-based sensing, especially in scenarios where visual information is limited. Furthermore, Flak and Czyba [17] developed an RF-based drone detection system using a distributed sensor grid with hardware-accelerated signal processing, showing strong robustness to lighting and weather variations. Although multi-modal solutions provide complementary information, vision-based video detection remains a critical component due to its intuitive interpretability and compatibility with existing surveillance infrastructures. However, detecting low-slow-small UAVs in complex backgrounds still poses significant challenges, which motivates the development of more robust and efficient video-based detection methods, as addressed in this work.

In this paper, we conclude from the case of human eyes searching for flying mosquitoes that [18] the human eye’s detection of extremely small targets mostly begins with being attracted by their motion characteristics, and is then followed by identification. In fact, there are certain similarities between flying mosquitoes and low-slow-small UAVs [19]: first, their size and pixel proportions are very small; second, the object can be easily submerged in the background texture.

Based on this, this paper introduces a novel low-slow-small UAV target detection method that integrates spatio-temporal information. The contributions of this paper are presented as follows:

(1) We propose a spatio-temporal framework for low-slow-small UAV target detection. It combines static detection and dynamic judgment for UAV detection, which enables effective UAV detection in complex scenarios and challenging conditions. Experimental results have shown superior performance to existing methods.
(2) We improve YOLOv11 static detection methods for fine-grained information extraction by firstly using SPD-Conv to preserve target details and BiFPN for cross-layer weighted feature fusion, and then adding a detection header based on shallow high-resolution layers to improve small UAV detection, which significantly enhances the performance of static image detection.
(3) We propose a dynamic target detection algorithm. It firstly aligns consecutive frames via LK optical flow, least squares, and RANSAC, and then the connected regions are extracted based on frame difference and morphological operations, which are input into the YOLO backbone for feature extraction and target identification, and finally, the bounding box is optimized through bilinear interpolation to improve target localization.
(4) The algorithm was implemented on an RK3588 embedded system for air-to-air UAV target detection. The quantitative results demonstrate that the proposed approach achieves Precision, Recall, and mAP50 metrics of 94%, 92%, and 86.3%, which significantly outperforms extant methods. Furthermore, through lightweight optimization of the core algorithm during deployment, the system attains processing with 100 frames per second, satisfying stringent real-time operational constraints.

This paper is organized as follows: Section 2 reviews the related work in video object detection, with a focus on recent advances in temporal and spatial methods. Section 3 presents our proposed methodology, including the model architecture. Section 4 describes the experimental setup, dataset, training process, and visualization experiments. Section 5 discusses the results, and Section 6 concludes the paper with future directions.

## 2. Related Work

### 2.1. Single-Frame Image-Based UAV Target Detection

One study used a boosted classifier cascade to test Haar-like features, the Histogram of Oriented Gradients (HOG), and Local Binary Patterns (LBP) for LSS UAV target detection [20]. Additionally, another study employed HOG features with the Adaboost algorithm for the online detection of LSS UAV targets [21]. Further research utilized two-dimensional, rotation-, and translation-invariant Generic Fourier Descriptor (GFD) features, classifying targets as drones or birds via a neural network [22].

Yanlong Chang et al. [23], based on the YOLOv5 model, decoupled the classification and regression tasks in the detection head and optimized the loss function definition using Alpha-IoU, improving the model’s effectiveness in detecting small targets in drone aerial imagery. Furthermore, Zhiyong Qin et al. [24], based on the YOLOv7 model, enhanced tiny object detection capability by optimizing feature fusion, adding a small target detection layer and improving the loss function. Siyuan Liu et al. [25], based on the YOLOv5l model, added an ECA attention mechanism and Ghost module, achieving improved small target detection performance and model lightweighting. RTD-Net, proposed by Ye et al. [26], leverages both CNNs and Transformers to achieve real-time target detection. By integrating the Transformer’s ability to capture long-range contextual information, RTD-Net effectively addresses the challenges of occluded object recognition and overcomes the limitations of CNNs in global feature association. Qiu et al. [27] introduced YOLO-Air, a YOLO-based network. The architecture combines multi-scale feature fusion, customized anchor frames, and a high-resolution feature layer tailored to small-target features, to enable efficient and accurate small-target detection for UAVs.

### 2.2. UAV Target Detection via Spatio-Temporal Information Association

In recent years, several studies have investigated video object detection by explicitly modeling temporal information across frames. Wu et al. [28] proposed SELSA, which aggregates semantic features at the sequence level to enhance temporal consistency among detections in adjacent frames. Chen et al. [29] introduced MEGA, a memory-enhanced global–local aggregation framework that leverages both short-term and long-term temporal cues through a dedicated memory module. More recently, Zhou et al. [30] presented TransVOD, an end-to-end video object detection framework based on transformers, which employs spatio-temporal attention to jointly model spatial and temporal dependencies across video frames. Although these methods achieve improved performance on general video benchmarks, they typically involve complex temporal modeling and high computational overhead. As a result, their effectiveness may be limited when applied to low-slow-small UAV detection under cluttered backgrounds and low-contrast conditions. In contrast, our method is specifically designed to balance temporal modeling capability and computational efficiency for such challenging scenarios.

To address these issues, Zhu et al. [31] proposed aggregating features from nearby frames via optical flow to enrich the feature representation of each frame. Wu et al. [32] proposed using whole sequence-level feature aggregation to improve video object detection. Chen et al. leveraged global semantic and local localization information from a memory aggregation network, achieving impressive results on video object detection benchmarks. Wu H X et al. [33] proposed MotionRNN to address the challenge of predicting spatio-temporal varying motions in videos, designing motion-aware dynamic loop units, combining the spatio-temporal attention mechanism, focusing on motion-significant regions and correlating with the key history frames letters to achieve accurate prediction of complex motions.

Motion R-CNN [34], based on frame difference methods, provides auxiliary information for small objects, improving their detection performance in optical remote sensing videos. DogFight proposed a two-stage segmentation method using spatio-temporal attention cues, where objects are localized in the first stage and tracked/filtered using temporal information in the second stage [35]. LSTFE-Net proposed a plug-and-play spatio-temporal feature alignment module to establish temporal correspondences between short-term frames and the current frame [36]. It then uses a frame selection module to choose long-term frames providing the most additional contextual information. Finally, the network fuses long- and short-term features through a long-short-term feature aggregation module. LSTFE-Net achieved excellent results on the FL-Drones dataset.

## 3. Method

### 3.1. Problem Statement

**Figure 2:**
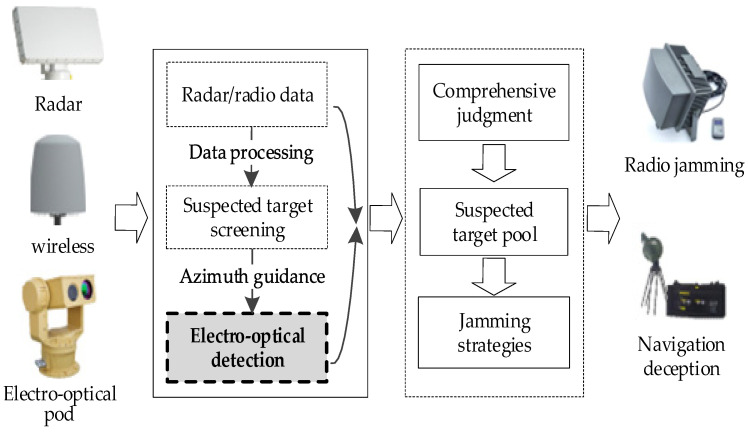
Typical UAV detection and countermeasure system.

As shown in Figure 2, the photoelectric detection module is positioned after the wide-area sensing and data processing stages, and is mainly responsible for target confirmation and re-identification. This design allows coarse detection results to be further verified using high-resolution visual information, thereby reducing false alarms and improving discrimination capability in low-slow-small UAV detection systems [37].

**Figure 3:**
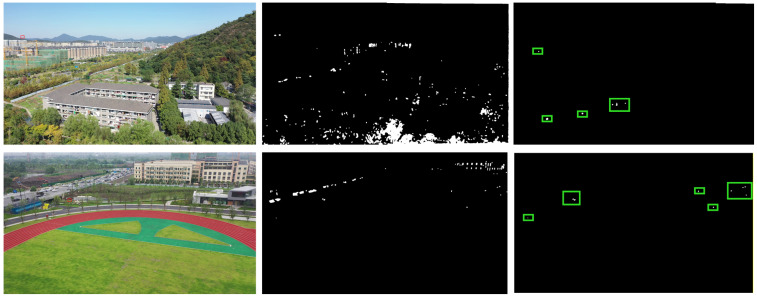
Motivation of the proposed method: UAVs are often indistinguishable from complex backgrounds in a single frame; inter-frame differencing effectively suppresses static background components and amplifies sparse moving responses, facilitating rapid candidate ROI extraction (green boxes).

By observing numerous LSS drone videos, we reveal similar patterns. We conclude that the detection of very small targets often begins by being attracted to their motion characteristics, followed by secondary identification. Figure 3 shows the original image, background-subtracted image, and its binarized image. Obviously, it is very challenging to detect UAVs from a single image frame, but through a simple dynamic frame difference process, the suspected target area is quickly screened out. And this is the foothold of the method proposed in this paper.

Furthermore, during downsampling operations like convolution and pooling in deep learning networks, the features of small-pixel targets will be gradually lost and fail to transfer to deeper layers. Taking YOLOv11 as an example, the original image is typically resized to 640 × 640 as an input and then downsampled by factors of 1/8, 1/16, and 1/32 in the backbone network. We assume an original image size of 1920 × 1080 pixels, with a UAV target size of 30 × 20 pixels. The effective target pixels become only 10 × 6 in the input, and the target resolution at the last stages of the backbone is less than 1 × 1 pixel. Figure 1 illustrates the target size during downsampling in the YOLOv11 backbone network. It is clear that the target information becomes very weak after 1/8 downsampling.

Based on this, we propose a method fusing dynamic information and static detection, including dynamic region screening, static target detection and the fusion strategy. Then, we will provide a detailed introduction of each scheme as follows.

### 3.2. Overall Detection Scheme

**Figure 4:**
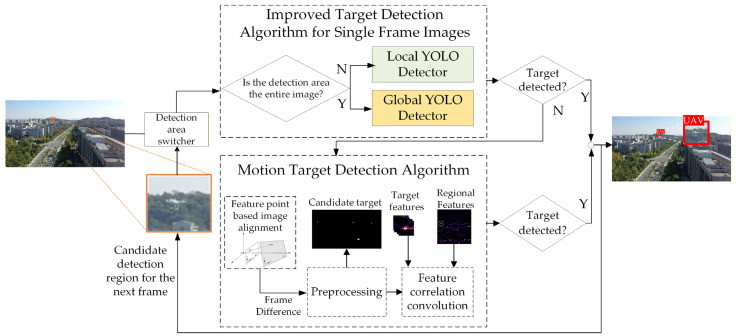
Overall framework of the proposed MSM-YOLO for low-slow-small UAV detection, including the improved YOLO-based detector, the motion-based module, and the static–dynamic fusion strategy.

The overall detection strategy is shown in Figure 4. Assume it is the input image at time *t*. We firstly perform an improved single-frame detection on *I_t_* using a high confidence threshold screening strategy (e.g., confidence threshold *α* ≥ 0.9) to suppress the false alarm rate.

Our algorithm has three main modules: an improved YOLO detector on original images, a region-based YOLO detector for candidate areas, and a motion target detection algorithm. The process starts with global detection using the original YOLO; if no target is found, motion detection is triggered. Once a target is detected, a candidate region for the next frame is generated based on past motion data, and the region-scale YOLO performs.

Moreover, when processing the *I_t_*_+1_ frame, the detected region will be dynamically switched based on the previous frame’s detection state. When a target is detected, a local area in frame *I_t_*_+1_ will be extracted and regarded as input. Assuming (*xt*,* yt*) is the object coordinate, we take the coordinates as center to generate a local region *R_t_* with 160 × 160 pixels.

If there is no target detected, the motion information will be activated into judgment, which can balance the detection efficiency and completeness. Specifically, the detection region selection strategy can be formalized as:

(1) $$R_{t+1}=\left\{\begin{array}{ll} \mathrm{ROI}(x_{t},y_{t},80,80) & \mathrm{if}\;D(I_{t})\neq \emptyset \\ I_{t+1} & \mathrm{otherwise} \end{array}\right.$$

where *D* (*·*) is the detection function, and ROI (*·*) is the generation function of the interest region, which is built as the center of (*x_t_*,* y_t_*) with a side length of 80 pixels.

**Figure 5:**
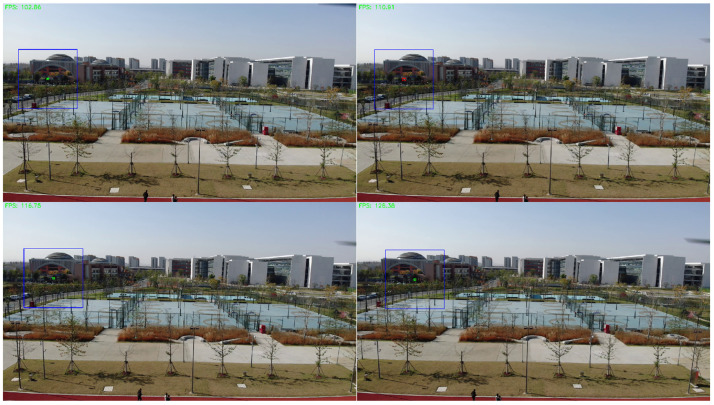
Representative frames show that when the UAV is hardly separable in a single image, inter-frame differencing yields sparse moving responses within the candidate ROI, enabling motion-assisted confirmation; when static cues remain reliable, the fusion prioritizes appearance evidence to suppress motion-induced false alarms. Red, blue, and green bounding boxes denote motion response regions, candidate regions, and final detection results, respectively.

Figure 5 qualitatively demonstrates the adaptability of the proposed reliability-aware spatio-temporal fusion. In scenes where the UAV is extremely small and barely distinguishable from the background in a single frame, the appearance-based detector may become uncertain or intermittently fail. In such cases, the motion branch is activated, and inter-frame differencing produces sparse and localized responses around the moving target, which provides complementary evidence to recover missed detections and maintain temporal continuity. Conversely, when the target is sufficiently visible and the static confidence is high, the system relies primarily on the appearance cues to avoid unnecessary motion-triggered false alarms. This adaptive switching behavior enables the method to handle varying target visibility and background complexity without introducing heavy computation, thereby supporting stable performance under diverse operating conditions.

### 3.3. Motion Detection Algorithm

**Figure 6:**
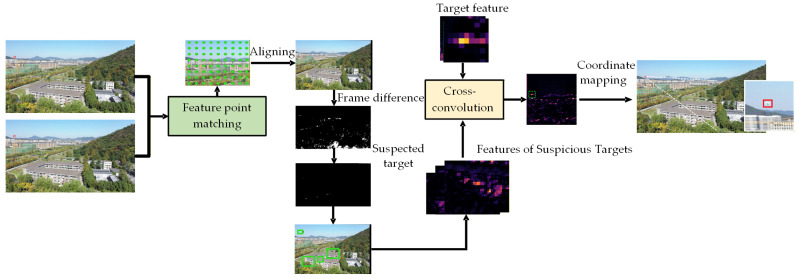
Pipeline of the motion-based target detection module: Our approach aligns consecutive frames using LK optical flow, least squares, and RANSAC to create a perspective transformation matrix and align neighboring frames, generates motion masks by frame difference and adaptive thresholding, and refines them by morphology. The connectivity domain is then extracted, fed into YOLO for feature extraction and inter-convolution with UAV template features, which are then mapped back to the original image and the bounding box for target localization is refined by bilinear interpolation.

The motion detection algorithm provides dynamic localization for target by analyzing pixel changes between consecutive frames [38]. As shown in Figure 6, we designed an adaptive moving target detection framework to calculate the micro-motion features of UAVs in complex backgrounds.

Assuming *I_t_* and *I_t_*_+1_ are two consecutive frames, we conduct the adaptive moving target detection by the next modules:

**Step 1 Grid Point Displacement:** Constructed a uniformly distributed grid of points *P_t_* (grid spacing set as *s* pixels). We explore the Lucas–Kanade (LK) optical flow algorithm to compute the displacement vectors *V_t_* of grid points between adjacent frames.

**Step 2 Homography Estimation:** Used least squares to estimate the homography matrix **H**_t_ and establish the perspective transfer relationship between the two frames:

(2) $$H=\arg\underset{H}{\min}\sum_{i=1}^{N}{\left\|p_{i,t+1}-H\cdot p_{i,t}\right\|}^{2}$$

where *P_t_* and P*_t_*_+1_ are the coordinates of grid points in frame *t* and *t* + 1. And the RANSAC algorithm will also be used to remove outlier matches and optimize the homography matrix estimation accuracy.

**Step 3 Image Alignment:** Utilized the estimated homography matrix *H_t_* to warp *I_t_* to align with *I_t_*_+1_, obtaining the aligned image *I_t’_*:

(3) $${\hat{I}}_{t+1}(x,y)=I_{t+1}(H^{-1}\cdot {\left(x,y,1\right)}^{T})$$

**Step 4 Frame Difference & Mask Generation:** Calculated the aligned frame difference image Difft = |*I_t_*_+1_ − *I_t_*|, and then generated a preliminary motion mask *M* via adaptive threshold:

(4) $$M(x,y)=\left\{\begin{array}{c} 1,\;\Delta I(x,y)>T(x,y)\\ 0,\;\;\;\mathrm{otherwise} \end{array}\right.$$

where *T*(*x*,* y*) is a dynamic threshold based on local pixel statistics (e.g., the sum of local mean and twice the standard deviation).

**Step 5 Morphological Processing:** Applied the morphological opening operation to filter noise and the closing operation to fill holes within targets, obtaining the optimized motion mask *M′*.

**Step 6 Candidate Region Extraction and Feature Matching:** For each connected region *C_i_* in *M′*, we extract the corresponding image sub-region *R_i_* via its bounding box and then input *R_i_* into the YOLO backbone network to extract feature map *F_i_*.

Let the target template feature be *T* (normalized from high-confidence samples), and then calculate the feature correlation between candidate regions and the template using cross-correlation:

(5) $$C_{k}=f_{\mathrm{temp}}⋆F_{k}=\sum_{i=1}^{D}\sum_{j=1}^{H}\sum_{k=1}^{W}f_{\mathrm{temp}}(i)\cdot F_{k}(i,j,k)$$

where ⋆ denotes 3D cross-correlation, and *D*, *H* and *W* are the channel number, height and width of feature maps. We then conduct target candidate region *R_k_* with the highest correlation score *C_k_*.

**Step 7 Coordinate Mapping and Refinement:** Let the candidate region’s coordinates of the feature map be (*xf*,* yf*). The downsampling stride of the feature map relative to the original image is *s*, and then we can map back to the original image coordinates:

(6) $$x=x_{f}\cdot s,\;y=y_{f}\cdot s,\;h=h_{f}\cdot s,\;w=w_{f}\cdot s$$

Finally, we adjust the bounding box accuracy by using bilinear interpolation to obtain the final target localization $\hat{B}=(x,y,h,w)$ of the original image.

### 3.4. Static Weak Small Target Detection Module

**Figure 7:**
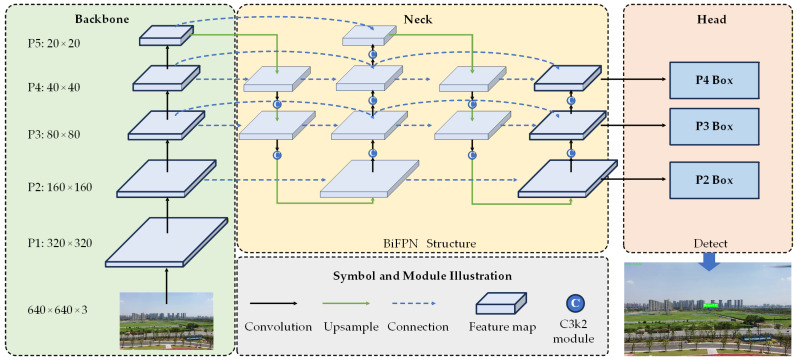
Improved static target detection algorithm based on YOLOv11.

We use the improved YOLO detection method for static target detection. However, since YOLO is limited in challenge scenarios such as small targets and complex backgrounds, this paper makes the following improvements aimed at increasing the usable small target feature information. The improved YOLOv11-based detection method is illustrated in Figure 7, which includes first introducing a SPD-Conv module to halve the spatial resolution while multiplying channel dimension and preserving the total information volume, and then using the BiFPN module to improve feature fusion and enhance the flow of shallow features via feature weighting, and finally redesigning the multi-scale branches with resolutions of 160 × 160, 80 × 80, and 40 × 40.

1. SPD Convolution Module (SPD-Conv)

SPD-Conv [39] is a novel Convolutional Neural Network (CNN) building block composed of a Space-to-Depth (SPD) layer and a non-stride convolution layer. It aims to solve the problem of fine-grained information loss caused by traditional stride convolution and pooling operations, particularly suitable for low-resolution images and small target detection tasks. SPD-Conv achieves efficient feature extraction through two key steps: the SPD layer and the non-stride convolution layer.

Specifically, the SPD layer downsamples the spatial dimensions of the input feature map while retaining all information in the channel dimension. For example, for an input feature map *I* of size *S* × *S* × *C*1, the SPD layer partitions it into *scale*^2^ sub-feature maps (e.g., generating four sub-maps when scale = 2), each of size (*S/scale*) × (*S/scale*) × *C*1. These are then concatenated along the channel dimension to obtain an intermediate feature map *X′* of size (*S/scale*) × (*S/scale*) × (*scale*^2^ × *C*1).

The non-stride convolution layer applies a convolution operation with stride = 1 after the SPD layer, compressing the channel count from *scale*^2^ × *C*1 to *C*2 (*C*2 < *scale*^2^ × *C*1), while preserving spatial detail information. This design avoids an asymmetric sampling issue of traditional stride convolution, ensuring the integrity of the feature representation.

2. Feature Fusion Architecture based on BiFPN

Due to the low resolution and weak semantic information, the traditional feature fusion architectures are difficult to effectively handle small targets in complex scenes. Therefore, this paper introduced the BiFPN (Bidirectional Feature Pyramid Network) [40] for feature fusion of the YOLO network. It introduces weighted feature fusion across layers, aligning semantics and details from different levels to enhance the distinction between target and background, and fuses high-level semantics with low-level details through bidirectional pathways, which significantly improve the feature discriminability for small targets.

The BiFPN module is an efficient multi-scale feature fusion architecture proposed by Google in EfficientDet. Specifically, BiFPN integrates bidirectional cross-scale connections: a top-down branch and a bottom-up branch. The top-down branch propagates high-level semantic features to lower levels via upsampling, enhancing semantic information for small targets. The bottom-up branch propagates low-level detail features to higher levels via downsampling, supplementing detail information for large targets. Additionally, cross-layer skip connections add extra edges at the same level, reusing original features to avoid information loss. Crucially, BiFPN incorporates weighted feature fusion, dynamically assigning the contribution of features from each level.

3. Detection Heads based on High-Resolution Feature Maps

Generally speaking, in CNN networks, the shallow feature maps retain higher spatial resolution due to fewer downsampling steps, and each pixel corresponds to a smaller local region of the original image, which captures detailed information like edges and textures. In contrast, for deep feature maps, each pixel has a large receptive field, which dilutes small target features and has a low resolution. However, small targets often occupy less than 32 × 32 pixels in an image, and their location-sensitive features are highly dependent on resolution. Solely relying on traditional three-scale detection heads (e.g., 80 × 80, 40 × 40, 20 × 20 in YOLOv11) will easily lead to missed detection.

This paper introduces detection heads at shallow, high-resolution feature layers (160 × 160, 80 × 80, 40 × 40). The experimental results show significant outcomes in small target detection. Combined with the BiFPN, the new detection header can fuse features of adjacent levels, which maintains high resolution while injecting some mid-level semantics, and forming a feature representation characterized by detail perception and local context [41].

## 4. Experiment

This section presents the evaluation of experiments for the proposed method. We provide the detailed datasets, evaluation metrics, experimental implementations and analysis.

### 4.1. Datasets

To evaluate the effectiveness of the proposed algorithm, we first tested it on the public challenging ARD-MAV datasets [42], and then established an experimental platform for real-world verification.

**Figure 8:**
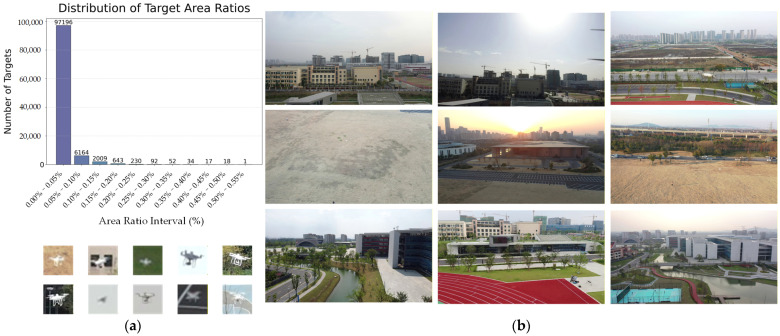
ARD-MAV dataset overview: (**a**) plot of statistical target size distribution for the ARD-MAV dataset; (**b**) the dataset has a wide variety of scenarios and most of the UAV targets have less than 0.05% of pixels.

The ARD-MAV datasets contain 60 video sequences and 106,665 frames of visible images, with a resolution of 1920 × 1080, and contain a wide variety of backgrounds such as mountains, buildings and sky. It also includes challenging conditions such as occlusion, sudden camera movement, fast-moving and extremely small UAVs. As shown in Figure 8, we provide the statistical results of UAV pixel proportion. It can be observed that the pixel proportion of the majority of UAV targets is less than 0.05%.

In addition to ARD-MAV, we further evaluate the proposed method on a mixed real-world dataset composed of the public M3D-real [43] subset and our self-collected UAV sequences. Specifically, the M3D-real sequences provide publicly available real-world UAV data with diverse outdoor scenes and challenging capture conditions, while our self-collected sequences complement M3D-real by introducing additional variations in camera platforms, background dynamics, and target scales. By merging these two sources, we construct an extended real-world testbed to conduct supplementary experiments, aiming to provide additional evidence of effectiveness and robustness beyond a single benchmark.

### 4.2. Evaluation Metrics

In this paper, we employ the Average Precision (AP) with an Intersection over Union (IoU) threshold set to 50%, and Precision, Recall, and F1-Score for experimental evaluations.

Precision represents the proportion of actual positive samples among all samples predicted as positive by the model:

(7) $$Precision=\frac{TP}{TP+FP}$$

where *TP* is True Positive and *FP* is False Positive.

Recall represents the proportion of samples predicted as positive by the model among all actual positive samples:

(8) $$Recall=\frac{TP}{TP+FN}$$

where *FN* is False Negative.

AP (Average Precision) is a comprehensive performance evaluation metric that effectively reflects the model’s performance under different thresholds. The Precision–Recall (P-R) curve is plotted with Precision on the *x*-axis and Recall on the *y*-axis. The integral of the P-R curve for each category is the AP for that category; a higher value indicates better performance.

Assuming the interpolated Precision–Recall curve is *P*(*r*), where *r* is the Recall rate, then the *AP* is the integral of this curve over the interval [0, 1].

(9) $$AP=\int_{0}^{1}P(r)dr$$

### 4.3. Experimental Details

As shown in Table 1, all experiments were performed on a Windows 11 computer equipped with an Intel Core i7-9700 CPU, 8 GB RAM, and an NVIDIA GeForce RTX 4060 GPU with 8 GB of VRAM.

For the static detection, we trained the model with an input of a 640 × 640 resolution image. The trained epochs are set to 60, with a batch size of 8. For the dynamic object classifier, the input image size is set to 32 × 32 and the number of trained epochs to 100 with a batch size of 32. Additionally, we utilized tensor RT and pycuda API to accelerate inference speed.

### 4.4. Evaluation Results Across Scenarios

We conducted performance evaluations of the proposed detection algorithm on a test set, which comprises sky, mountain and building background. Based on the complexity of the scene background, we first classify scenarios for analysis.

**Figure 9:**
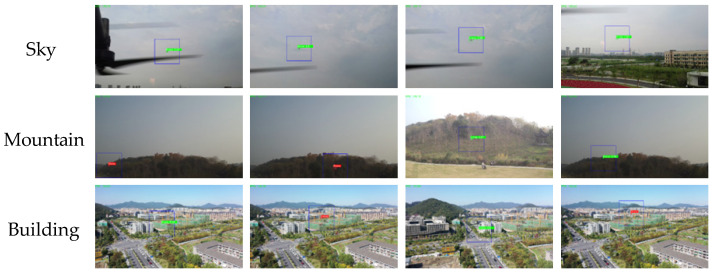
Comparison of the detection results of the MSM-YOLO algorithm in different scenarios; each line represents the output of different scenarios.

As shown in Table 2, the algorithm demonstrates excellent performance in the sky background, which achieves Precision of 0.98, Recall of 0.95, F1-Score of 0.96 and mAP50 of 0.892. While in the mountain background, disturbed by various different interference, Recall drops to 0.67, yet still maintains a high Precision of 0.96, and with the F1-Score and mAP50 as 0.79 and 0.732, respectively. Moreover, for the building background, the proposed method achieves Precision of 0.93, Recall of 0.83, F1-Score of 0.88 and mAP50 of 0.783, which is demonstrated to be adapted for multiple scenarios. Figure 9 shows some instances of UAV detection results, which indicate the proposed method successfully detects UAV under various conditions, such as clear identification of UAV against the sky background, effective capture of UAV in mountainous environments, and precise localization of UAV within building areas, which verifies the robustness of the algorithm in diverse scenarios.

### 4.5. Comparative Experiments

To better evaluate the proposed method, Table 3 lists the quantitative comparison of the proposed MSM-YOLO with several state-of-the-art methods on various datasets. All baselines were trained with the same epochs and image size, and no method was tuned using the test set. As shown in Table 3, the proposed method excels across multiple metrics, and significantly outperforms the existing advanced methods, which indicates that the existing methods exhibit significant deficiencies in small UAV object detection, leading to substantial decreases in Recall, F1-Score and AP.

Specifically, when the UAV size is as small as 20 × 20 pixels in a 1920 × 1080 image, the target pixel proportion is only 0.019%. Moreover, since the targets are constantly moving and changing within complex environments, just using a single-frame detecting method is obviously insufficient. In contrast, considering the continuity of object motion characteristics, the proposed MSM-YOLO method, on one hand, generates a candidate region of 160 × 160 pixels in the next frame from the detection of the previous frame, and on the other hand, adds motion features for judgment when static detection fails. Furthermore, when the UAV is obscured by the background and remains undetected for several consecutive frames, the motion target detection algorithm within MSM-YOLO detects the UAV and generates a candidate detection region for the next frame, which supplements the overall detection accuracy. The experimental results effectively demonstrate that the combined use of dynamic and static information can significantly reduce interference from complex backgrounds and thereby greatly enhance detection performance.

**Figure 10:**
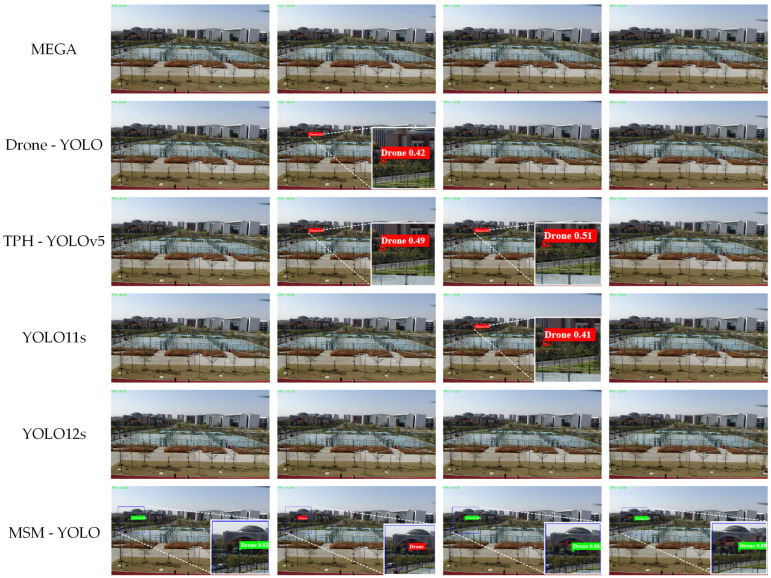
Comparison of typical detection result samples achieved by various methods, with each row presenting the output of a different detection method.

As shown in Figure 10, the proposed MSM-YOLO method demonstrates outstanding performance in detecting UAV targets. On one hand, most UAV targets are effectively identified through the local detection strategy, while on the other hand, in some complex challenge scenes, such as the fourth column of images in Figure 9, the motion target detection module in the MSM-YOLO method can effectively identify the correct target, whereas other methods fail to detect. Overall speaking, the experimental results demonstrate the effectiveness of the proposed MSM-YOLO algorithm for small UAV detection tasks.

In addition, Table 4 reports the quantitative comparison results on the mixed real-world dataset constructed from the M3D-real subset and our self-collected UAV sequences. As can be observed, the proposed MSM-YOLO achieves the highest Recall (0.871) and mAP@0.5 (0.924) among all compared methods, while maintaining a competitive Precision of 0.958. Compared with YOLOv8 + P2 and TPH-YOLOv5, MSM-YOLO consistently improves Recall, indicating a stronger ability to detect extremely small and fast-moving UAV targets under complex real-world conditions. Although FBRT-YOLO exhibits reasonable precision, its significantly lower Recall and mAP suggest limited robustness when target appearance is weak, or motion cues are subtle.

Notably, the superior Recall performance of MSM-YOLO demonstrates that the proposed spatio-temporal motion–static fusion strategy effectively alleviates the common issue of missed detections in small UAV scenarios. By leveraging motion continuity to generate candidate regions and activating motion-based detection when static appearance cues become unreliable, MSM-YOLO is able to recover targets that are easily overlooked by single-frame detectors. These results further confirm that incorporating temporal motion information is beneficial for improving detection robustness on real-world datasets with severe background interference and extremely small target scales.

### 4.6. Ablation Study on the Improved YOLO Detector

To verify the effectiveness and importance of each improvement strategy proposed in this paper, we conducted ablation experiments on the ARD-MAV datasets. The experimental results are shown in Table 5. As seen in Table 5, we provide a detailed experimental analysis of each improvement strategy, including adding the SPD convolution module, modifying the detection head, and feature fusion based on BiFPN.

**Analysis of Adding SPD Convolution Module:** To reduce the information loss of small UAV targets during model downsampling, this study incorporates the SPD convolution module. The SPD halves the spatial resolution while doubling the channel dimension, preserving the total information content and avoiding the defect of directly discarding pixels inherent in pooling or stride convolution. This ensures the fine-grained information of small targets is completely preserved. By adding the SPD convolution module, the method’s Precision, Recall, and mAP50, respectively, increased by 2%, 2.7%, and 1.4%, compared to the baseline. The results indicate the SPD module helps to improve the detector’s performance for small targets.

**Analysis of High-Resolution Detection Header:** Small UAV targets occupy a very small pixel proportion within an image. With multiple downsampling operations, the feature information becomes increasingly blurred. To effectively utilize the target’s feature information, this study adds a tiny object prediction header (P2) and removes the large object prediction header. Table 5 shows a comparison of the impact of different detection heads on detection accuracy. Compared to the baseline, by adding the prediction header for P2 feature layer, the model’s Precision, Recall, and mAP50 increased by 2.4%, 10.1%, and 9.2%, respectively. Furthermore, compared to only adding the P2 header, by adding the P2 feature layer and removing the 20 × 20 feature layer (large object header), the model’s Precision, Recall, and mAP50 increased by 0.3%, 0.7%, and 0.6%, respectively. The results indicate that the large target prediction header does not improve the model’s detection performance for small UAV targets and verifies the effectiveness of the 160 × 160 feature layer (P2) and the redundancy of the 20 × 20 feature layer for detecting UAV targets.

**Analysis of BiFPN Feature Fusion Module:** To validate the effectiveness of BiFPN for small UAV detection, this study focuses on feature utilization and propagation in complex scenes, addressing the limitations of traditional feature fusion architectures. BiFPN constructs a bidirectional feature fusion pathway, by strengthening the interaction of multi-scale hierarchical features, it achieves efficient integration of shallow fine-grained details (e.g., small target edges, textures) and deep semantic information, tackling the detection challenges caused by the small scale of targets and susceptibility to background interference. Using datasets covering diverse scenes like mountain buildings, square facilities and suburban landscapes, we compared the YOLO11 detection performance and attention distribution of the integrated BiFPN fusion module.

**Figure 11:**
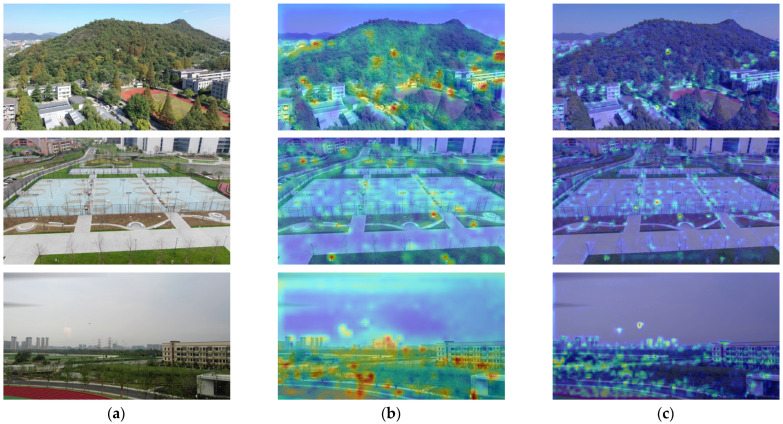
Heatmap visual analysis. (**a**) Original images. (**b**) Without BiFPN. (**c**) With BiFPN.

The visualization results are shown in Figure 11, which lists the results without BiFPN and with BiFPN. From the results, we observe that, without the BiFPN, the model’s attention distribution is scattered and blurred, while small target feature extraction is incomplete, and background interference cannot be effectively filtered, which leads to the detection Precision and Recall being suppressed. While with the BiFPN, its bidirectional fusion mechanism precisely integrates multi-scale features, focusing attention on the core target and suppressing redundant background, significantly optimizing feature utilization efficiency. Compared to the baseline model, the BiFPN fusion module further improved the detection performance, by which the improved YOLO model achieved a Precision, Recall, and mAP50 increase by 12.1%, 29.5% and 29.6%, with a better accuracy, fewer missed detections and similar parameter size. Moreover, with inference acceleration optimization, it meets the requirements for high-precision and real-time UAV detection.

### 4.7. Ablation Study of MSM-YOLO

In this section, we analyze the different components of MSM-YOLO to verify their effectiveness in UAV detection. The experimental results in Table 6 indicate the importance of each component of the proposed method. After combining the various algorithmic modules, the overall algorithm achieves Precision, Recall and mAP50 of 94%, 92% and 86.3%. While S-YOLO uses the improved YOLO for entire image detection, SM-YOLO uses the improved YOLO and the motion detection algorithm on the entire image, MS-YOLO uses the improved YOLO detector with a local candidate region for the next frame, and MSM-YOLO is the proposed method, which combines the use of the improved YOLO algorithm both globally and in candidate regions, and the motion target detection algorithm. The experimental analysis of each module is as follows:

**Motion Target Detection Algorithm:** The experimental results in Table 6 show that the motion target detection algorithm significantly improves Recall compared to the simple YOLO based approaches. By capturing the subtle morphological changes in targets across consecutive frames, it compensates for the defect of easy missed detection by static detection styles in complex backgrounds and small target scenarios. When the YOLO detector struggles to distinguish targets from background, the motion target detection algorithm firstly identifies suspicious changed regions between consecutive frames and then relocates the target, which strengthens the ability for continuous moving target detection.

**Candidate Region Detection:** The experimental results in Table 6 demonstrate the local candidate region plays a crucial role compared to simply using YOLO on the entire image. Candidate region detection avoids the problem of small target pixel dilution caused by downsampling, since the proportion of target features decreases drastically when shrinking a large original image. By reducing background interference and focusing on the target’s historical motion cues, it assists in improving detection accuracy, which forms a small target detection optimization of detail enhancement and interference filtering. As shown in Table 6, compared to using YOLO on the entire image, the method incorporating the candidate region detection module improves Precision and Recall by 6.0% and 8.0%, respectively, indicating significant improvement.

**Figure 12:**
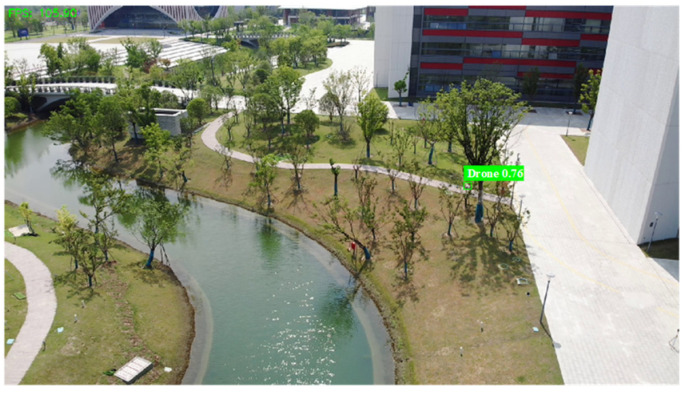
Improved YOLO detector on global images.

**Figure 13:**
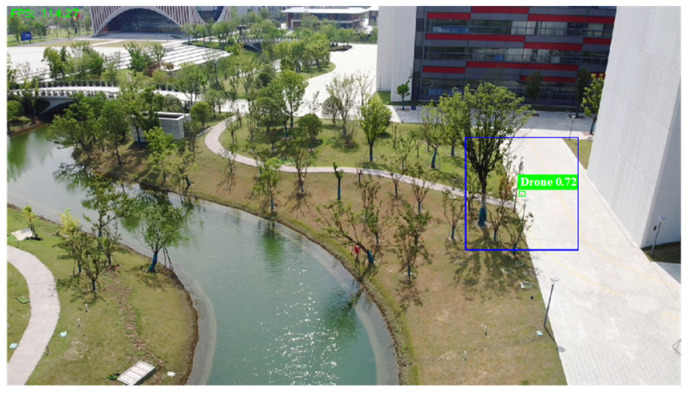
Improved YOLO detector on candidate detection regions.

**Figure 14:**
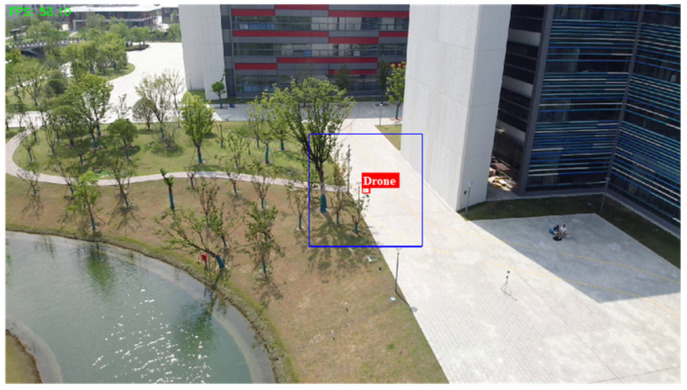
Motion target detection algorithm detects moving low-slow-small UAV targets.

**Multi-module Collaborative Detection:** MSM-YOLO combines the motion detection, candidate region detection and the improved YOLO detector. Through multi-module collaboration and the utilization of spatio-temporal information between consecutive frames, it achieves a better performance in small UAV detection. As shown in Figure 12, Figure 13 and Figure 14, the overall workflow of MSM-YOLO is as follows:

Firstly, an improved YOLO target detector is used for the entire frame detection, and then a candidate detection region for the next frame is generated and detection is performed on the candidate region. If the above methods fail, the motion detection algorithm is employed to supplement the overall detection, in which the candidate detection region is continuously updated. Ultimately, the proposed MSM-YOLO achieves Precision, Recall, and mAP50 of 94%, 92%, and 86.3%, enabling effective detection of small UAV targets in complex scenarios.

### 4.8. System Demonstration

**Figure 15:**
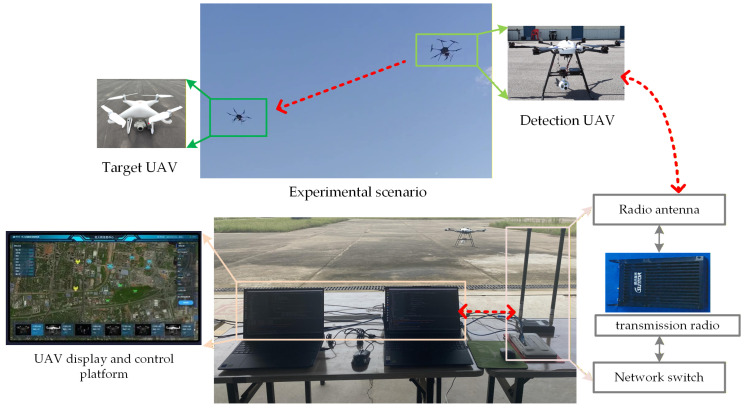
Design of the overall architecture.

To further verify the effectiveness of the proposed method, we deployed it on an airborne platform to conduct LSS UAV detection. Figure 15 proposed the overall architecture of the physical verification system, in which the detection UAV carries an electro-optical sensor that captures real-time videos, and the AI platform performs a real-time detection algorithm. The PC is used to control the UAV and data display, where the two platforms transmit data via a radio station. When detecting a target, the UAV transmits detection information to the PC terminal, which then displays the real-time images and detection results.

**Figure 16:**
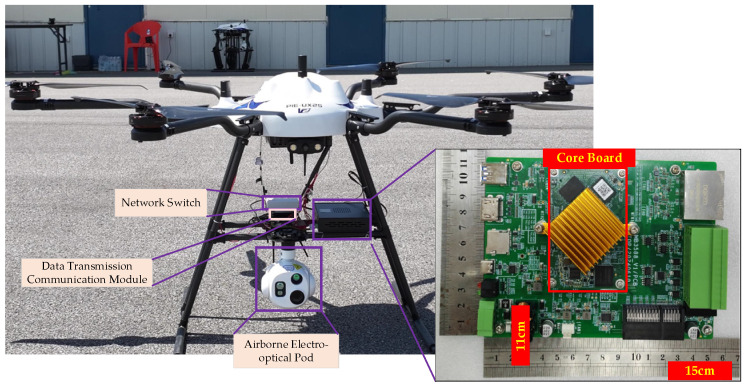
UAV detection experimental system.

To further verify the practicality of the proposed method, we select PIE-UX25 as the detection UAV, and select the DJI Mavic 3 and DJI Phantom 4 Pro UAV as the target UAV. The PIE-UX25 equips an electro-optical and an onboard RK3588 platform system, and has a maximum payload capacity of 10 kg. The entire system is shown in Figure 16, and Table 7 summarizes the key experimental conditions and system parameters used in the airborne embedded verification. RK3588 was selected as the main control chip. We redesigned the main control board based on RK3588 core chip, which has an actual size of 15 cm × 11 cm, and used for task management, information transmission and target detection. The designed RK3588 platform includes interfaces such as Ethernet and serial ports to connect with the pod, UAV controller and network radio.

**Figure 17:**
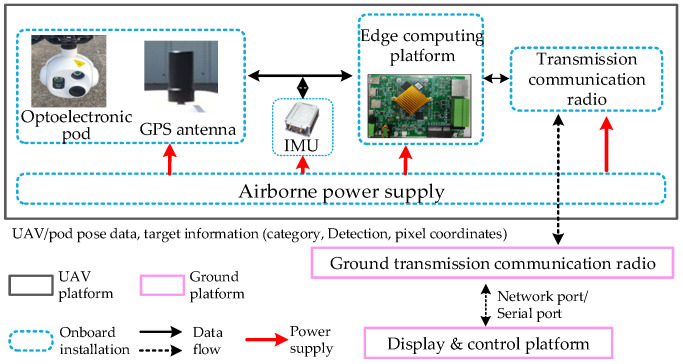
The detailed electrical interfaces and data flow interaction of UAVs.

The detailed data flow interaction of UAVs is shown in Figure 17. The RK3588 platform firstly obtains the image sensor data, and then conducts target detection, finally transmitting the detection information to the PC platform via radio station. Moreover, in order to achieve unified management, the onboard sensors and processing systems are powered by a unified power supply.

**Figure 18:**
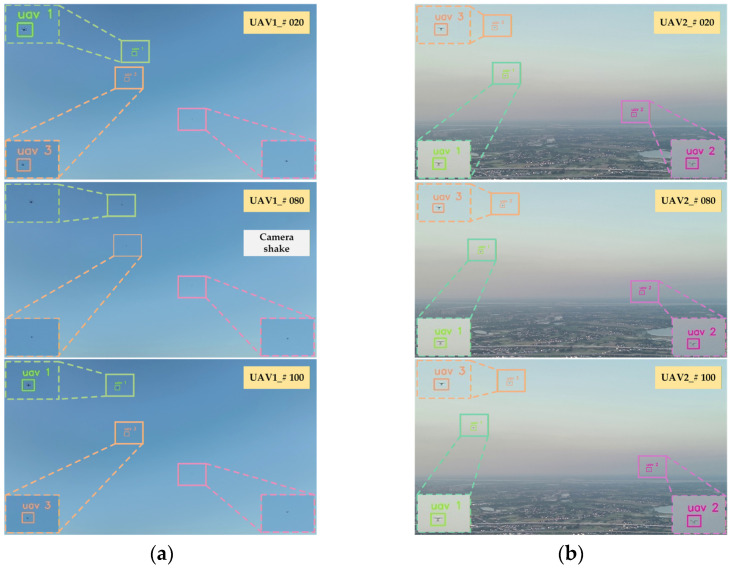
Air-to-air UAV object detection results. (**a**) UAV#1 detection viewpoint; (**b**) UAV#2 detection viewpoint.

Based on the thread pool management technology of RK3588, we accelerated the entire detection process, which involves pod video stream decoding and detection thread inference. Figure 18 shows the detection results of our algorithm for multiple UAV targets in an air-to-air scenario. The left side (a) shows the detection perspective of UAV #1, and the right side (b) shows the perspective of UAV #2. In the view of UAV #1, detection difficulty increases in some video frames due to environmental factors like sky background and lighting. The perspective of UAV #2 provides additional target and scene information from a different observation dimension. Even when the view from UAV #1 experiences temporary detection fluctuations due to shaking, the algorithm can still maintain effective detection of the target by relying on the detection results obtained from UAV #2. The detection performance across different perspectives fully verifies the reliable detection effectiveness of the algorithm in complex air-to-air scenarios, even when facing environmental interference (background, camera shake) and the challenge of identifying small UAV targets.

## 5. Discussion

This section summarizes the most significant quantitative findings and their implications for low-slow-small (LSS) UAV detection. First, the improvements to the YOLO11 baseline lead to clear gains in small-target detection, with the best enhanced variant achieving +29.6% in mAP50, +29.5% in Recall, and +12.1% in Precision. These results indicate that strengthening high-resolution feature representation and multi-scale fusion is essential to address the extremely small target size and the susceptibility to cluttered backgrounds, thereby reducing missed detections.

Second, adding spatio-temporal components further improves performance. Compared with S-YOLO (the improved YOLO for entire image detection), the complete MSM-YOLO achieves +15.9% mAP50, +31% Recall, and +11% Precision. The most pronounced gain is observed in Recall, suggesting that motion-guided cues and the integrated static–dynamic fusion strategy effectively recover hard-to-detect targets and improve temporal consistency. Notably, with lightweight optimization during deployment, the method achieves around 100 FPS, indicating that the accuracy improvements can be obtained without sacrificing real-time efficiency.

Overall, the results confirm that combining a stronger spatial baseline with lightweight temporal modeling provides a robust and efficient solution for LSS UAV detection.

## 6. Conclusions

In this paper, we proposed an LSS UAV target detection algorithm combining spatio-temporal information, which has been proven effective in challenging conditions such as complex backgrounds and extremely small targets. The proposed algorithm primarily consists of three modules: (1) an improved YOLO detector for small UAV detection, (2) a motion target detection module, and (3) an integrated combination strategy of static and dynamic information. The experimental results on UAV datasets have shown the effectiveness of the proposed algorithm, which contains diverse scenarios with varying scales of UAV targets and complex background changes. Furthermore, we deployed the proposed method on an airborne RK3588 platform for real-world UAV detection, demonstrating its practical feasibility and preliminary real-scene performance. However, the proposed approach still has limitations. Its performance may degrade in highly cluttered backgrounds or very low target-background contrast scenarios, and the motion-based module is dependent on the quality of inter-frame displacement estimation, which can be affected by severe camera jitter or motion blur. In future work, we will research cross-domain target detection for UAVs in multi-scenario settings to improve the algorithm’s generalization. We will also investigate multi-modal fusion perception models to achieve high-precision perception of small UAV targets. In addition, we will conduct large-scale field validation with systematic task-level quantitative metrics to further assess real-world performance under more diverse conditions.

## Figures and Tables

**Table 1:** Experimental platform configuration.

Experimental Environment	Versions
Programming Languages	Python 3.11
Deep Learning Framework	Pytorch 2.0.0
CUDA	11.7
OS	Windows 11
GPU	NVIDIA RTX 4060 (8 GB)

**Table 2:** Comparative results in different background scenarios.

Conditions	Precision	Recall	F1-Score	AP50
Sky	0.98	0.95	0.96	0.892
Mountain	0.96	0.67	0.79	0.732
Building	0.93	0.83	0.88	0.783

**Table 3:** Comparative results with peer methods on the ARD-MAV dataset.

Method	Precision	Recall	F1-Score	mAP50
MEGA [29]	0.45	0.35	0.39	0.310
Drone-YOLO [44]	0.82	0.29	0.43	0.432
TPH-YOLOv5 [45]	0.87	0.27	0.41	0.465
YOLO11s	0.81	0.36	0.50	0.424
YOLO12s	0.80	0.27	0.41	0.354
Ours	**0.94**	**0.92**	**0.93**	**0.863**

**Table 4:** Comparative results with peer methods on the M3D-Real dataset.

Method	Precision	Recall	mAP50
YOLOv8 + P2	0.921	0.816	0.897
TPH-YOLOv5 [45]	**0.959**	0.82	0.875
FBRT-YOLO [46]	0.867	0.628	0.733
Ours	0.958	**0.871**	**0.924**

**Table 5:** Ablation study results on individual modules in YOLO11.

Model	Model Size	Precision	Recall	F1-Score	mAP50	mAP50-95
Yolo11	5.5 M	0.752	0.338	0.466	0.424	0.224
Yolo11 + SPD	20.4 M	0.772	0.365	0.496	0.438	0.233
Yolo11 + P2	5.7 M	0.776	0.439	0.561	0.516	0.267
Yolo11 + P2H3	**4.2 M**	0.779	0.446	0.567	0.522	0.268
Yolo11 + P2H3 + SPD	15.1 M	0.839	0.543	0.659	0.620	0.334
Yolo11 + P2H3 + SPD + BiFPN	14.2 M	**0.873**	**0.633**	**0.734**	**0.720**	**0.387**

**Table 6:** Ablation study results on individual modules.

Method	Precision	Recall	F1-Score	mAP50
S-YOLO	0.83	0.61	0.70	0.704
SM-YOLO	0.85	0.68	0.76	0.735
MS-YOLO	0.89	0.69	0.78	0.751
MSM-YOLO	**0.94**	**0.92**	**0.93**	**0.863**

**Table 7:** Experimental conditions and PIE-UX25 multirotor UAV system parameters.

Experimental Conditions	Parameters
Maximum Load	10 kg
Maximum Flight Speed	15 m/s
Maximum Take-Off Weight	30 kg
Maximum Endurance	70 min

## Data Availability

The datasets used in this study are available at https://github.com/WindyLab/Global-Local-MAV-Detection (accessed on 28 January 2026) and https://github.com/WindyLab/M3D (accessed on 28 January 2026).

## Abbreviations

The following abbreviations are used in this manuscript:

UAV	Unmanned Aerial Vehicle
FOV	Field of view
LK	Lucas–Kanade
HOG	Histogram of Oriented Gradients
LBP	Local Binary Patterns
GFD	Generic Fourier Descriptor
LSS	Low-Slow-Small
CNN	Convolutional Neural Network
IoU	Intersection over Union
